# Knowledge, Attitudes, and Practices Regarding Antibiotic Use and Resistance Among the General Adult Population in Riyadh Province, Saudi Arabia

**DOI:** 10.7759/cureus.80392

**Published:** 2025-03-11

**Authors:** Nemer A Alotaibi, Abdulrahman M Alshahrani, Saad M Alsaab, Fahad M Aldehaim, Sultan S Aldalbahi, Mohammad A Rashikh, Mohammad I Ahmad

**Affiliations:** 1 Pediatrics, College of Medicine, Shaqra University, Dawadmi, SAU; 2 Neurology, College of Medicine, Shaqra University, Dawadmi, SAU; 3 Internal Medicine, College of Medicine, Shaqra University, Dawadmi, SAU; 4 Medicine, College of Medicine, Shaqra University, Dawadmi, SAU; 5 Pharmacology and Therapeutics, College of Medicine, Shaqra University, Dawadmi, SAU; 6 Pharmaceutical Sciences, Nibha Institute of Pharmaceutical Sciences, Nalanda, IND

**Keywords:** antibiotic misuse, antibiotic resistance, antibiotics self-medication, general population, saudi arabia

## Abstract

Background

Antibiotic resistance has become a global concern, contributing to significant morbidity and mortality. The prominent cause of antibiotic resistance remains the overuse and inappropriate use of antibiotics. This study aimed to evaluate the knowledge, attitudes, and practices (KAP) regarding antibiotic use and antibiotic resistance among the general adult population.

Methodology

A cross-sectional survey was conducted among the general adult population using a self-administered, validated questionnaire with 21 questions. A chi-square test and binary logistic regression analysis were applied to recognize factors associated with KAP and antibiotic use.

Results

Out of 562 participants, the majority were female (408, 72.6%) and between the ages of 18 and 25 (489, 87%). The study findings revealed that most participants had poor knowledge (501, 89.1%), inadequate attitudes (405, 72.1%), and inappropriate practices (282, 50.2%) regarding antibiotic use. Misconceptions were common, with 360 (64%) participants believing antibiotics are effective against viral infections and 237 (42.2%) unaware that inappropriate use and incomplete antibiotic courses can lead to antibiotic resistance. The binary logistic regression analysis revealed that respondents with postgraduate and marital status were significant predictors of better knowledge and more appropriate attitudes about antibiotic use. A significant positive Spearman correlation was observed between participants’ KAP scores (P<0.001).

Conclusion

This study identified crucial gaps in KAP regarding antibiotic use among the population of Riyadh province. Our findings provide valuable insights for health policymakers to design targeted intervention programs for deficient groups to enhance the population’s KAP regarding the appropriate use of antibiotics.

## Introduction

Antibiotics are fundamental to treating infectious diseases; however, their overuse and misuse in recent decades have led to a rise in antimicrobial resistance (AMR). This escalation poses an increased risk of morbidity, mortality, and healthcare costs [1,2]. AMR is now a critical global health issue and an important human threat [3]. A recent study has reported that bacterial AMR was directly responsible for 1.14 million global deaths in 2021 and was associated with 4.71 million deaths [4]. If left unchecked, it is projected that by 2050, AMR could directly cause 1.91 million deaths and be associated with 8.22 million deaths globally [4]. Globally, the overuse and misuse of antibiotics are attributed to various factors, including the availability of antibiotics as over-the-counter medications, the tendency to store leftover antibiotics for future use, insufficient patient education, misuse of antibiotics for viral infections, failure to complete prescribed courses, the overuse of broad-spectrum antibiotics [5-10]. Antibiotic self-medication (ASM) prevalence is estimated at 58% in Asia, 47% in Southern Europe, 30% in Eastern Europe, 25% in South America, and 39% in the Middle East [11]. ASM practices contribute to AMR and adverse effects [5].

The public plays an important role in reducing antibiotic misuse [12]. Several countries have implemented national campaigns to address public misconceptions about antibiotics, encourage appropriate use, and prevent the development of AMR [13-19]. Only a few studies have evaluated the general population's knowledge, attitudes, and practices (KAP) regarding antibiotic use and resistance in Saudi Arabia [6,20,21]. This study aimed to evaluate the KAP towards antibiotic use among the general adult population in Riyadh province, Saudi Arabia.

## Materials and methods

Study design

The cross-sectional observational study was conducted in Riyadh Province, Saudi Arabia, from November 28, 2023, to February 1, 2024. The inclusion criteria were (a) the general adult population of Saudi Arabia; (b) both genders, male and female; and (c) an age ≥18 years. The exclusion criteria were as follows: (a) working in the health sector as a doctor, nurse, pharmacist, or dentist; and (b) hospitalized patients.

Sample size

OpenEpi software (Centers for Disease Control and Prevention (CDC), Atlanta, USA) and the formula $n = \frac{z^2 \cdot p \cdot (1 - p)}{e^2}$ were used to calculate the sample size, where n represents the sample size, Z is the level of confidence (95% confidence interval (CI) = 1.96), p is the prevalence of ASM (63.4%) [22], and e is the margin of error (5%). The minimum recommended sample size (n) for this study was 357.

Data collection tools and validity

The validated questionnaire used in this research was adapted from previous similar studies [19,23-25]. It was translated into Arabic and validated through a pretest involving 20 Arabic-speaking participants, including students and senior researchers. The pretest feedback from participants confirmed that the questionnaire was clear, comprehensible, and easy to complete. The electronic data collection links (Google Forms) were distributed to the target study population through WhatsApp, Telegram, and email. The cover letter of the questionnaire tool emphasized the study’s objectives and inclusion criteria. The convenience non-probability sampling method was employed for this study. Participants’ consent was obtained before they began the questionnaire, and they were allowed to proceed or opt out of the study. The questionnaire was structured into four sections. Section I included demographic data with five questions. Section II evaluated knowledge of antibiotic use and antibiotic resistance through nine questions. Section III evaluated attitudes towards antibiotic use with six questions, while Section IV examined practices related to antibiotic use through six questions. The reliability coefficient of responses to the final questionnaire was assessed using Cronbach’s alpha score, and the following results were noted: knowledge (0.688), attitudes (0.645), and practices (0.682). A Cronbach’s alpha of more than or equal to 0.6 is generally considered acceptable for internal consistency [26,27].

Statistical analysis and data management

Data were analyzed using IBM SPSS (Statistical Package for the Social Sciences) software version 25.0.0.0 (SPSS Inc., Armonk, NY, United States). Descriptive statistical analysis was conducted to express each characteristic as frequencies and percentages for categorical variables. A chi-square test and binary logistic regression analysis were employed to identify demographic factors associated with KAP. Results are reported as odds ratios (ORs) and 95% CIs. The p-values <0.05 were considered statistically significant. Spearman's correlation coefficient was used to identify the strength and direction of the relationship between responses to the questions about KAP.

One point was assigned for each correct response for the KAP scores and zero for incorrect or not-sure responses. The antibiotic KAP score was calculated by adding the participant’s correct responses across 21 statements. The scales were constructed as follows: a knowledge scale (0 to 9), an attitude scale (0 to 6), and a practice scale (0 to 6) based on the number of correct answers. KAP scores were categorized as “good” or “poor” using an 80% cut-off. A score ≤80% (1-7 for knowledge, 1-4 for attitude and practice) was classified as “poor,” while a score of 81%-100% (8-9 for knowledge, 5-6 for attitude and practice) was classified as “good.”

Ethical approval

The study protocol received ethical approval from the Institutional Ethics Committee at Shaqra University (HAPO-01-R-128) under approval number ERC_SU_S_202300011.

## Results

Characteristics of the respondents

Five hundred seventy-seven questionnaires were distributed, achieving an impressive 568 (98.4%) responses. Among these, 562 participants met the inclusion criteria. A clear majority of respondents, 489 (87%), were aged 18 to 25 and were female (408, 72.6%). A total of 457 (81.3%) completed their undergraduate education, while 17 (3.1%) were postgraduates. Most participants, 457 (81.3%), reported a monthly income below 5000 Saudi riyals (Table 1).

**Table 1:** Socio-demographic characteristics of study participants (N=562) SAR: Saudi Riyal

Variables		Frequency (N)	Percentage (%)
Age group (in years)	18-25	489	87.0
	26-35	34	6.0
	36-45	18	3.2
	≥46	21	3.9
Gender	Male	154	27.4
	Female	408	72.6
Educational level	Primary school	3	0.5
	Secondary or high school	85	15.1
	Graduate	457	81.3
	Postgraduate	17	3.1
Marital status	Single	496	88.3
	Married	66	11.7
Monthly income (in SAR)	≤5000	457	81.3
	>5000	105	18.7

Responses to questions regarding KAP of antibiotic use

Regarding knowledge, when participants were asked about using specific medications as antibiotics, 257 (45.7%) agreed that amoxicillin is an antibiotic, 205 (36.5%) identified Augmentin as an antibiotic, and 312 (55.5%) correctly disagreed that paracetamol is not an antibiotic. A total of 397 (70.6%) correctly acknowledged that antibiotics effectively treat bacterial infections. However, 360 (64%) of participants had misconceptions about the role of antibiotics in treating viral infections, and 332 (59.1%) incorrectly agreed with their use for most coughs, common colds, and flu. Regarding accessibility, 403 (71.7%) of participants disagreed with the statement that antibiotics can be purchased without a prescription. Over half of the participants, 325 (57.8%), correctly agreed that inappropriate use and failure to complete the entire course of antibiotics can lead to antibiotic resistance. However, 398 (70.8%) participants were unaware of antibiotic resistance.

Regarding attitude, 337 (60%) participants displayed a positive attitude by not sharing antibiotics with family or friends, and 400 (71.2%) trusted their doctor’s decision not to prescribe antibiotics. Additionally, 466 (82.9%) followed the instructions on the label before using antibiotics. However, 214 (38.1%) reported using antibiotics when experiencing symptoms of a cold or flu, 258 (45.9%) discontinued antibiotics before completing the course when they felt better, and 266 (47.3%) believed antibiotics speed up disease recovery.

In terms of practice, 207 (36.8%) used leftover antibiotics for repeated illness, 178 (31.7%) bought antibiotics from pharmacies without a doctor’s prescription, and 181 (32.2%) obtained antibiotics from relatives or friends. However, more than two-thirds, 390 (69.4%), always checked the expiration date of antibiotics before use, and 353 (62.8%) changed the dose only after consulting a doctor. Furthermore, 271 (48.2%) reported never using antibiotics when experiencing a fever (Table 2).

**Table 2:** Responses to questions regarding knowledge, attitudes and practices of antibiotic use (n=562) n: frequency; %: percentage

Questions		Correct Response	Agree % (n)	Disagree % (n)	Neutral % (n)
Knowledge	Amoxicillin is used as an antibiotic.	Agree	45.7 (257)	4.1 (23)	50.2 (282)
	Augmentin is used as an antibiotic.	Agree	36.5 (205)	8.2 (46)	55.3 (311)
	Paracetamol (Panadol) is used as an antibiotic.	Disagree	32.4 (182)	55.5 (312)	12.1 (68)
	Antibiotics are used to treat bacterial infections.	Agree	70.6 (397)	14.1 (79)	15.3 (86)
	Antibiotics are used to treat viral infections.	Disagree	47.1 (265)	36 (202)	16.9 (95)
	Antibiotics are used to cure most coughs, common colds and flu.	Disagree	59.1 (332)	30 (169)	10.9 (61)
	Antibiotics can be purchased from the pharmacy without a prescription.	Disagree	17.3 (97)	71.7 (403)	11 (62)
	Inappropriate use and failure to complete the entire course of antibiotics can lead to antibiotic resistance.	Agree	57.8 (325)	14.6 (82)	27.6 (155)
	Antibiotic resistance is a phenomenon where drug potency diminishes, making it more challenging to kill pathogens.	Agree	29.2 (164)	8.2 (46)	62.6 (352)
Attitudes	I always share my leftover antibiotics with family and friends when they are sick with the same symptoms.	Disagree	22.9 (129)	60 (337)	17.1 (96)
	I most often use antibiotics when experiencing symptoms of a cold or flu.	Disagree	38.1 (214)	46.6 (262)	15.3 (86)
	I stop taking antibiotics before completing the course when I feel better.	Disagree	45.9 (258)	38.4 (216)	15.7 (88)
	I trust the doctor when he decides not to prescribe antibiotics.	Agree	71.2 (400)	9.6 (54)	19.2 (108)
	I use antibiotics according to the instructions on the label.	Agree	82.9 (466)	6.7 (38)	10.3 (58)
	I usually take antibiotics to speed up disease recovery.	Disagree	47.3 (266)	29.7 (167)	23 (129)
Practices	I use leftover antibiotics for repeated illness.	Disagree	36.8 (207)	58.9 (331)	4.3 (24)
	I always check the expiration date before using the antibiotics.	Agree	69.4 (390)	20.3 (114)	10.3 (58)
	I usually change the dose of antibiotics without consulting a doctor.	Disagree	19.7 (111)	62.8 (353)	17.4 (98)
	I take antibiotics when I have an illness with a fever.	Disagree	34.9 (196)	48.2 (271)	16.9 (95)
	I usually get antibiotics from relatives or friends.	Disagree	32.2 (181)	63.9 (359)	3.9 (22)
	I usually buy antibiotics from the pharmacy without a doctor’s prescription.	Disagree	31.7 (178)	56.6 (318)	11.7 (66)

KAP scores of study participants

**Figure 1:**
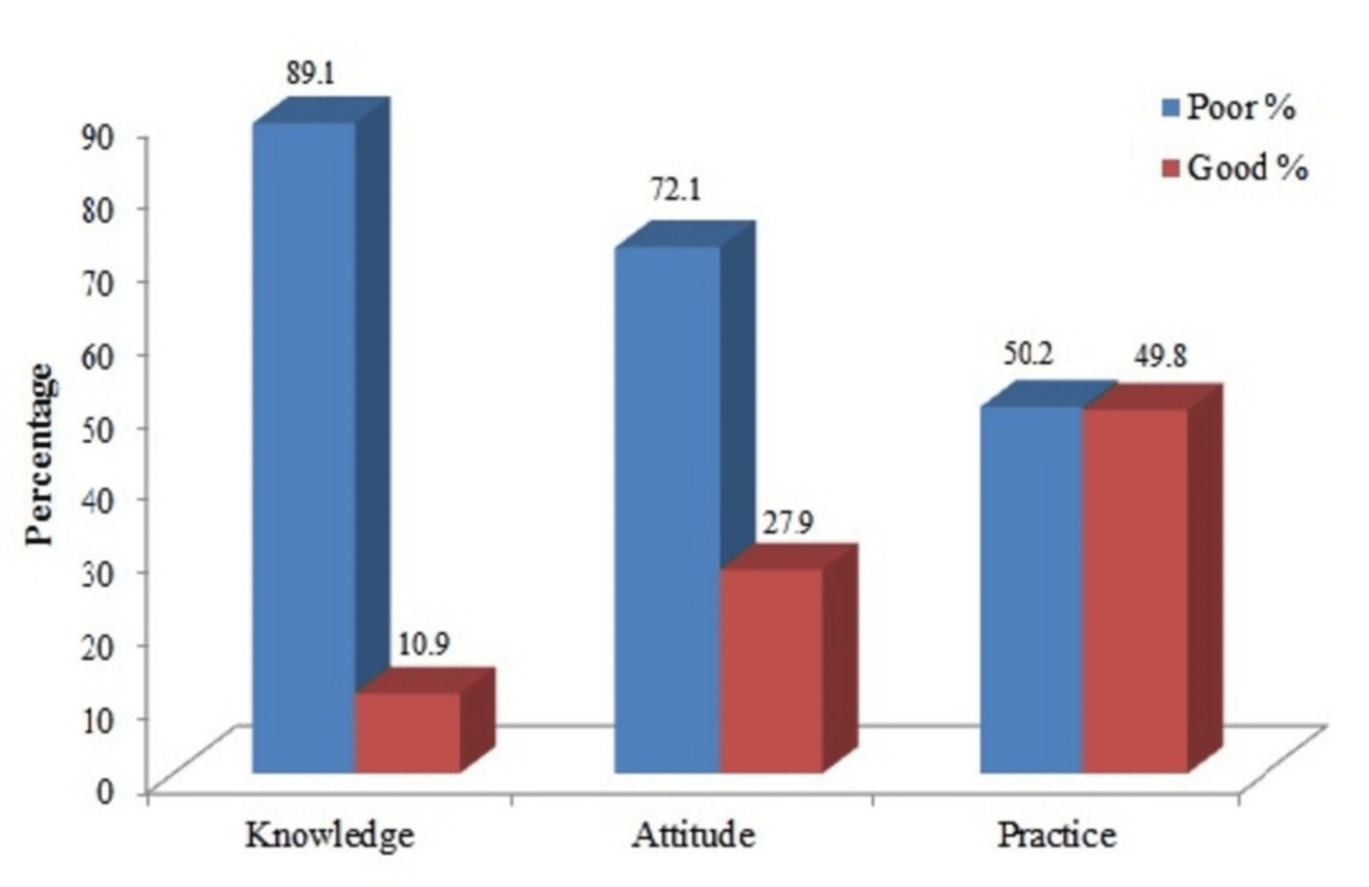
Overall KAP score levels regarding antibiotic use and resistance Score levels ≤80% = poor KAP, score levels 81-100% = good KAP KAP: knowledge, attitudes, and practices

Only 10.9% of participants demonstrated good knowledge scores, 27.9% showed good attitudes, and 49.8% exhibited good practices regarding antibiotic use (Figure 1).

Participant characteristics associated with KAP scores relating to antibiotics use

Regarding knowledge, people with higher educational levels (χ2 = 12.873; p-value = 0.005) and marital status (χ2 = 21.033; p-value = 0.013) exhibited significantly better knowledge than those with lower educational and single social statuses.

Regarding attitude, people with higher educational levels (χ2 = 8.083; p-value = 0.044), advanced aged group (χ2 = 16.171; p-value = 0.001) and marital social status (χ2 = 14.319; p-value = 0.026) revealed significantly higher scores of positive attitude than people with lower educational levels, younger age groups and single social status (p<0.05).

Regarding practices, people of younger age, lower educational level, and higher income displayed more inappropriate practices than people of advanced age, higher educational level, and lower income status. However, no significant associations were observed with sociodemographic variables (p>0.05) (Table 3).

**Table 3:** Participant characteristics associated with KAP scores relating to antibiotics use (n=562) KAP: knowledge, attitudes, and practices; SAR: Saudi Riyal n: frequency; %: percentage; p-values were generated using the Persian chi-square (χ2) test for comparing two groups. Significant at p-value ≤0.05.

Variables		% (n)								
		Knowledge score			Attitudes score			Practices score		
		Poor (≤7) (n=501)	Good (>7) (n=61)	χ^2^ (p-value)	Poor (<5) (n=405)	Good (≥5) (n=157)	χ^2^ (p-value)	Poor (<5) (n=282)	Good (≥5) (n=280)	χ^2^ (p-value)
Age (in years)	18-25	89.9 (440)	10.1 (49)	4.652 (0.199)	74.4 (364)	25.6 (125)	16.171 (0.001)	51.3 (251)	48.7 (238)	2.222 (0.528)
	26-35	88.2 (30)	11.8 (4)		67.6 (23)	32.4 (11)		44.1 (15)	55.9 (19)	
	36-45	83.3 (15)	16.7 (3)		55.6 (10)	44.4 (8)		44.4 (8)	55.6 (10)	
	≥46	76.2 (16)	23.8 (5)		38.1 (8)	61.9 (13)		38.1 (8)	61.9 (13)	
Gender	Female	90.7 (370)	9.3 (38)	3.651 (0.056)	73.5 (300)	26.5 (108)	1.588 (0.208)	50.5 (206)	49.5 (202)	0.058 (0.810)
	Male	85.1 (131)	14.9 (23)		68.2 (105)	31.8 (49)		49.4 (76)	50.6 (78)	
Level of education	Primary school	100 (3)	0 (0)	12.873 (0.005)	100 (3)	0	8.083 (0.044)	66.7 (2)	33.3 (1)	0.563 (0.905)
	High School	84.7 (72)	15.3 (13)		65.9 (56)	34.1 (29)		50.6 (43)	49.4 (42)	
	Graduate	90.8 (415)	9.2 (42)		74 (338)	26 (119)		50.3 (230)	49.7 (227)	
	Postgraduate	64.7 (11)	35.3 (6)		47.1 (8)	52.9 (9)		41.2 (7)	58.8 (10)	
Marital status	Single	90.5(449)	9.5 (47)	21.033 (0.013)	74.4 (369)	25.6 (127)	14.319 (0.026)	50.6 (251)	49.4 (245)	27.958 (0.262)
	Married	78.8 (52)	21.2 (14)		54.5 (36)	45.5 (30)		46.9 (31)	53.1 (35)	
Monthly income (in SAR)	≤5000	89.7(410)	10.3 (47)	11.438 (0.247)	73.5 (336)	26.5 (121)	5.089 (0.533)	49.5 (226)	50.5 (231)	25.562 (0.376)
	>5000	86.7 (91)	13.3 (14)		65.7 (69)	34.3 (36)		53.3 (56)	46.7 (49)	

Binary logistic regression analysis of factors influencing KAPs of antibiotics use

Regarding knowledge, people with a postgraduate education were approximately five times less likely (OR = 0.203, CI = 0.072-0.568, p‐value = 0.002) to have inaccurate knowledge compared to people with the lowest levels of education. Additionally, married people were approximately three times less likely (OR = 0.389; CI = 0.201-0.754, p‐value = 0.005) to have improper knowledge about antibiotic use than single people. No significant differences were observed regarding age group, gender, and monthly income.

Regarding attitudes, people aged 26-35 years were approximately five times less likely (OR = 0.211; CI = 0.086-0.552, p‐value = 0.001) to have improper attitudes about antibiotic consumption compared to people of ages 18-25. Similar results were observed for educational level; postgraduate people were three times less likely (OR = 0.353; CI = 0.137-0.911, p‐value = 0.031) to have improper attitudes compared to people with the lowest level of education. Regarding marital status, married people were approximately half as likely (OR = 0.413; CI = 0.244-0.698, p‐value = 0.001) to have improper attitudes than single people. However, no significant differences were observed regarding gender and monthly income. Regarding practices, no significant associations were observed with any sociodemographic variables concerning antibiotic use (p>0.05) (Table 4).

**Table 4:** Binary logistic regression analysis of factors influencing KAPs of antibiotics use among respondents Ref.: reference; KAP: knowledge, attitudes, and practices; OR: odds ratio; CI: confidence interval; SAR: Saudi Riyal; n: frequency; %: percentage; p-values were generated using the multivariable binary logistic regression analysis for comparing categorical values; significant at p-value ≤0.05.

Predictor		Knowledge score			Attitudes score			Practices score		
		OR	95% CI	p-value	OR	95% CI	p-value	OR	95% CI	p-value
Age group (in years)	18-25	Ref.	-	-	Ref.	-	-	Ref.	-	-
	26-35	0.356	0.125-1.015	0.053	0.211	0.086-0.552	0.001	0.584	0.115-1.547	0.240
	36-45	0.427	0.100-1.815	0.249	0.294	0.094-0.917	0.035	0.779	0.173-2.406	0.660
	≥46	0.640	0.130-3.155	0.583	0.492	0.137-1.772	0.278	0.769	0.207-3.020	0.688
Gender	Female	Ref.	-	-	Ref.	-	-	Ref.	-	-
	Male	0.585	0.336-1.019	0.058	0.771	0.515-1.156	0.208	0.955	0.659-1.384	0.810
Educational level	Primary school	Ref.	-	-	Ref.	-	-	Ref.	-	-
	Secondary or high school	0.001	0.000	0.999	0.001	0.000	0.999	0.400	0.030-5.248	0.485
	Graduate	0.366	0.117-1.150	0.085	0.527	0.189-1.473	0.222	0.800	0.288-2.226	0.669
	Postgraduate	0.203	0.072-0.568	0.002	0.353	0.137-0.911	0.031	0.793	0.307-2.046	0.631
Marital status	Single	Ref.	-	-	Ref.	-	-	Ref.	-	-
	Married	0.389	0.201-0.754	0.005	0.413	0.244-0.698	0.001	0.865	0.517-1.446	0.579
Monthly income (in SAR)	≤5000	Ref.	-	-	Ref.	-	-	Ref.	-	-
	>5000	0.745	0.393-14.11	0.366	0.690	0.439-1.086	0.109	1.168	0.764-1.787	0.474

Correlation between KAP

Spearman correlation revealed a positive association between each pair of the KAP scores for respondents (p<0.001) (Table 5).

**Table 5:** Correlations between knowledge, attitudes, and practices

Variables	Correlation coefficient	p-value
Knowledge - Attitudes	0.267	0.001
Knowledge - Practices	0.179	0.001
Attitudes - Practices	0.427	0.001

## Discussion

Antibiotic resistance is a pressing global issue, with its prevalence steadily increasing in Saudi Arabia, mainly due to rising cases of extended-spectrum *Escherichia coli* and *Klebsiella pneumonia* [28]. The Kingdom of Saudi Arabia, as the host of the annual Hajj, faces heightened concern over the growing threat of antibiotic resistance [29]. Consequently, evaluating the public’s KAP regarding antibiotic use and antibiotic resistance is crucial.

The present study findings revealed that 501 (89.1%) had poor knowledge scores regarding antibiotic use and antibiotic resistance. This prevalence is higher than the studies reported in Qatar (60%) and Kuwait (53%) [19,30]. This difference is primarily attributed to study setting variations, population, and included more educated people. Poor knowledge is a significant contributor to antibiotic misuse and the development of antibiotic resistance [31].

Regarding knowledge questions about the role of antibiotics, 397 (70.6%) participants correctly believed that antibiotics are effective in treating bacterial infections, a finding consistent with the study from Alkharj, Saudi Arabia (64.3%) [20]. However, this percentage is lower than that of studies from Bangladesh (96%) and Poland (80%) [23,32]. Conversely, more than half of the participants (360, 64%) held the misconception that antibiotics are effective in treating viral infections. This belief is higher than the studies from Romania (37.5%), Indonesia (12.9%), and the western region of Saudi Arabia (51.1%) [6,33,34].

A total of 332 (59.1%) study participants inappropriately believed that antibiotics effectively cure the common cold, most coughs, and flu. This finding aligns with a study from Lebanon (56.1%) but is higher than the United States (35%) findings [8,35]. These studies highlight a lack of understanding among participants who may struggle to differentiate between bacterial and viral infections, mistakenly believing that antibiotics can treat both. Such misperceptions contribute to the suboptimal use of antibiotics. Therefore, it is essential to enlighten the public that viral infections, including the common cold and flu, do not respond to antibiotic treatment.

In the current study, 403 (71.7%) respondents correctly stated that antibiotics should not be purchased from a pharmacy without a doctor’s prescription. However, this appropriate knowledge is lower than findings from Bangladesh (93%) [23]. Additionally, 325 (57.8%) of participants understood that inappropriate use and failure to complete the entire course of antibiotics could lead to antibiotic resistance, a proportion lower than studies from Indonesia (67.7%) [34]. Furthermore, only 164 (29.2%) participants were familiar with antibiotic resistance, a phenomenon where drug potency diminishes, making it more challenging to kill pathogens. This finding is considerably lower than those from Bangladesh (90%) and the Aseer region of Saudi Arabia (44%) [23,24]. This low level of knowledge about antibiotic use and antibiotic resistance underscores the need for policymakers to implement robust initiatives to raise public awareness across Saudi Arabia.

We also examined individual attitudes toward antibiotic use, with 157 (27.9%) respondents demonstrating a good attitude in this study. This frequency is significantly lower compared to a previous study from Qatar (60.2%) [30]. By demographics, participants in the advanced age group (>46 years) and married individuals exhibited better positive attitudes, aligning with findings from studies in Bangladesh and Qatar [23,30]. Additionally, those with higher education levels exhibited better positive attitudes toward antibiotic use, consistent with findings from a previous study in Saudi Arabia [25]. These findings underscore the need for targeted awareness campaigns, specifically aimed at those with lower educational levels and those living single, to promote the appropriate consumption of antibiotics.

In the present study, 129 (22.9%) participants shared leftover antibiotics with family members and friends, a negative attitude higher than reported in Romania [33]. These differences may be attributed to the socioeconomic status of the study population. Regarding adherence to completing antibiotic treatment, 258 (45.9%) respondents did not complete their last prescribed course, stopping treatment because they felt better. This finding is consistent with studies from Qatar (45%) but higher than those from Romania (29.2%) and Kuwait (36%) [19,30,33]. This high prevalence of negative attitudes likely reflects participants’ lack of knowledge about the risks of not completing antibiotic courses. It is well-established that inadequate dosing and incomplete treatment contribute significantly to antibiotic resistance [32].

Our results show that 214 (38.1%) respondents held inappropriate attitudes toward antibiotic use when experiencing cold or flu symptoms. This finding aligns with a study from Romania (30.1%) [33]. A total of 266 (47.3%) participants believed that using antibiotics speeds up disease recovery, reflecting a higher rate of negative attitudes than in a study in Indonesia [34]. Conversely, 400 (71.2%) participants expressed trust in doctors when they decided not to prescribe antibiotics. This finding is comparable to a study in Kuwait [19].

Of the total participants, 280 (49.8%) exhibited good practices regarding antibiotic use. This finding is significantly lower than those reported in Romania (80.1%) [33]. A total of 207 (36.8%) participants reported using the leftover antibiotics for repeated illnesses, aligning with studies from Qatar (27%) and Indonesia (31%) [30,34]. According to WHO guidelines, leftover antibiotics should never be used or shared to prevent the spread of AMR [36]. One hundred and ninety-six (34.9%) people admitted to taking antibiotics for any illness associated with a fever, similar to findings from Romania, Indonesia, and Makkah [33,34,37].

Regarding ASM, 181 (32.2%) participants received antibiotics from relatives or friends, a percentage similar to findings in Indonesia (35%) and Kuwait (27.5%) [19,34]. Additionally, 178 (31.7%) respondents preferred to purchase antibiotics from pharmacies without a doctor’s prescription, a proportion lower than the study in Indonesia (41%) [34].

Regarding KAP scores of antibiotics use, participants in the advanced age group (>46 years), those who were married, postgraduates, and male exhibited higher KAP scores. These findings align with the studies from Qatar and Italy [30,38]. Moreover, the present study found no significant association between KAP scores and monthly income groups, consistent with findings from Romania [33]. Our study concluded that education affects knowledge levels while income does not. This could be attributed to Saudi Arabia’s policy of providing all citizens free access to higher education.

The study identified crucial gaps in KAPs regarding antibiotic use, providing baseline information for future evaluations and the development of targeted educational interventions in Saudi Arabian communities. Based on these findings, future campaigns in Saudi Arabia should focus on six key messages: (1) the risks of self-medication; (2) the ineffectiveness of antibiotics against viral infections such as the common cold and influenza; (3) antibiotic resistance phenomenon and its consequences; (4) the importance of completing the entire course of antibiotics as prescribed by clinicians; (5) avoiding the storage and reuse of antibiotics for similar purposes; and (6) the requirement to purchase antibiotics only with a doctor’s prescription.

Limitations

The present study has certain limitations. First, the study population was a convenience sample, which may not fully represent the diverse populations of Saudi Arabia. The study received higher responses from the female gender, younger age, and low-income population. Second, the cross-sectional nature of this study makes it difficult to interpret cause-effect relationships. Future research should include longitudinal studies, larger sample sizes, and face-to-face questionnaires to assess the effects of antibiotic awareness on antibiotic usage.

## Conclusions

The study findings revealed that most participants demonstrated inadequate knowledge, negative attitudes, and inappropriate practices regarding antibiotic use, as evidenced by the low percentage of good KAP scores. More than half of the participants held misconceptions about the effectiveness of antibiotics against viruses or bacteria. People with low educational levels, single social status, and female gender were observed to have lower knowledge, poorer attitudes, and inappropriate practices compared to their peers. These findings provide valuable insights for health policymakers to design targeted intervention programs for deficient groups. The study recommends continuous awareness campaigns to promote the rational use of antibiotics and enhance public understanding of antibiotic resistance. However, further detailed research on the KAP of the general population is essential to ensure societal progress in addressing antibiotic misuse and antibiotic resistance.
